# Awake prone positioning on diaphragmatic function: Really bad or maybe good?

**DOI:** 10.1186/s13054-021-03866-1

**Published:** 2021-12-29

**Authors:** Basang Xiao, Yuntao Zhang, Zhuoga Ci, Dandan Shi, Hangyong He

**Affiliations:** 1 Department of Pulmonary Medicine, Lhasa People’s Hospital, Lhasa, Tibet Autonomous Region China; 2 Department of Respiratory and Critical Care Medicine, Beijing Institute of Respiratory Medicine, Beijing Chao-Yang Hospital, Capital Medical University, No. 8 Gongren Tiyuchang Nanlu, Chaoyang, Beijing, 100020 China

We thank Dr. Xiao and colleagues for their interest in our investigation [1] and for giving us the opportunity to further discuss our findings. They argue that a rise in diaphragmatic thickening fraction, consequent to awake prone position application (APP), may be a protective effect in COVID-19 acute hypoxemic respiratory failure (ARF).

Non-invasive respiratory support (NIRS) [6], in combination or not to APP, has been employed to stabilize the respiratory status and avoid intubation during COVID-19 outbreak, characterized by elevated intensive care unit surge capacity. Prone position improves gas exchange, lung aeration, and survival, mainly in intubated patients undergoing sedation and paralysis. In delivering NIRS, it is of pivotal importance to assure patient’s comfort, mainly when the non-invasive assistance is continuously delivered many hours per day as in COVID-19-related ARF. This is even truer with APP, when patients are requested to continuously lay in a prone obligated position.

An increase in the electrical activity of diaphragm, an index of diaphragmatic activation during assisted breath, has been previously reported in concomitance of a reduction in comfort in post-extubation NIRS patients [7]. Also, APP has not been able to reduce spontaneous inspiratory effort in all the patients from a cohort of subjects intubated for moderate-to-severe ARDS not related to COVID-19 [8]. Thus, proning awake patients seems more challenging than applying prone position or anterior chest compression in sedated and paralyzed patients undergoing invasive mechanical ventilation. This is easily understood considering that NIRS delivered for many hours a day, in combination with the APP, requires the continuous optimization of the patient-ventilator interaction, as well as the patient's full cooperation and tolerance in maintaining the prone position. In this condition, the advanced respiratory monitoring tools, i.e., diaphragm ultrasound, electrical impedance tomography, and esophageal pressure, might be a valid option to identify, early, patients at risk for self-induced lung injury.

Diaphragm ultrasound has been extensively employed to assess diaphragmatic activity in patients undergoing NIRS, yet on admission to the emergency department [9]. To date, however, data on diaphragmatic thickening fraction assessment in course of APP are scarce. Accordingly, the real impact of diaphragm ultrasound in this setting needs to be addressed in larger and multicenter investigations.

In conclusion, the increased diaphragmatic thickening fraction could also be the consequence of an improved lung aeration induced by APP, provided that this occurs without comfort deterioration. Therefore, APP's success is a simple compromise, as always.

## Data Availability

Not applicable.

## References

[1] Cammarota G, Rossi E, Vitali L, Simonte R, Sannipoli T, Anniciello F, Vetrugno L, Bignami E, Becattini C, Tesoro S (2021). Effect of awake prone position on diaphragmatic thickening fraction in patients assisted by noninvasive ventilation for hypoxemic acute respiratory failure related to novel coronavirus disease. Crit Care.

[2] Kaur R, Vines DL, Mirza S, Elshafei A, Jackson JA, Harnois LJ, Weiss T, Scott JB, Trump MW, Mogri I (2021). Early versus late awake prone positioning in non-intubated patients with COVID-19. Crit Care.

[3] Patel BK, Wolfe KS, Pohlman AS, Hall JB, Kress JP (2016). Effect of noninvasive ventilation delivered by helmet vs face mask on the rate of endotracheal intubation in patients with acute respiratory distress syndrome: a randomized clinical trial. JAMA.

[4] Carteaux G, Tuffet S, Mekontso Dessap A (2021). Potential protective effects of continuous anterior chest compression in the acute respiratory distress syndrome: physiology of an illustrative case. Crit Care.

[5] Marini JJ, Gattinoni L (2021). Improving lung compliance by external compression of the chest wall. Crit Care.

[6] Cammarota G, Esposito T, Azzolina D, Cosentini R, Menzella F, Aliberti S (2021). Noninvasive respiratory support outside the intensive care unit for acute respiratory failure related to coronavirus-19 disease: a systematic review and meta-analysis. Crit Care.

[7] Cammarota G, Longhini F, Perucca R, Ronco C, Colombo D, Messina A (2016). New setting of neurally adjusted ventilatory assist during noninvasive ventilation through a helmet. Anesthesiology.

[8] Yoshida T, Tanaka A, Roland R, Quispe R, Taenaka H, Uchiyama A (2021). Prone position reduces spontaneous inspiratory effort in patients with acute respiratory distress syndrome: a bicenter study. Am J Respir Crit Care Med.

[9] Cammarota G, Sguazzotti I, Zanoni M, Messina A, Colombo D, Vignazia GL (2019). Diaphragmatic ultrasound assessment in subjects with acute hypercapnic respiratory failure admitted to the emergency department. Respir Care.

